# A Versatile Technique for the *In Vivo* Imaging of Human Tumor Xenografts Using Near-Infrared Fluorochrome-Conjugated Macromolecule Probes

**DOI:** 10.1371/journal.pone.0082708

**Published:** 2013-12-17

**Authors:** Hiroshi Suemizu, Kenji Kawai, Yuichiro Higuchi, Haruo Hashimoto, Tomoyuki Ogura, Toshio Itoh, Erika Sasaki, Masato Nakamura

**Affiliations:** 1 Biomedical Research Department, Central Institute for Experimental Animals, Kawasaki, Japan; 2 Pathology Research Department, Central Institute for Experimental Animals, Kawasaki, Japan; 3 Animals Resource Department, Central Institute for Experimental Animals, Kawasaki, Japan; 4 Marmoset Research Department, Central Institute for Experimental Animals, Kawasaki, Japan; 5 Department of Applied Developmental Biology, Central Institute for Experimental Animals, Kawasaki, Japan; 6 Department of Pathology and Regenerative Medicine, Tokai University School of Medicine, Isehara, Japan; University of Pécs Medical School, Hungary

## Abstract

Here, we present a versatile method for detecting human tumor xenografts *in vivo*, based on the enhanced permeability and retention (EPR) effect, using near-infrared (NIR) fluorochrome-conjugated macromolecule probes. Bovine serum albumin (BSA) and two immunoglobulins—an anti-human leukocyte antigen (HLA) monoclonal antibody and isotype control IgG_2a_—were labeled with XenoLight CF770 fluorochrome and used as NIR-conjugated macromolecule probes to study whole-body imaging in a variety of xenotransplantation mouse models. NIR fluorescent signals were observed in subcutaneously transplanted BxPC-3 (human pancreatic cancer) cells and HCT 116 (colorectal cancer) cells within 24 h of NIR-macromolecule probe injection, but the signal from the fluorochrome itself or from the NIR-conjugated small molecule (glycine) injection was not observed. The accuracy of tumor targeting was confirmed by the localization of the NIR-conjugated immunoglobulin within the T-HCT 116 xenograft (in which the orange-red fluorescent protein tdTomato was stably expressed by HCT 116 cells) in the subcutaneous transplantation model. However, there was no significant difference in the NIR signal intensity of the region of interest between the anti-HLA antibody group and the isotype control group in the subcutaneous transplantation model. Therefore, the antibody accumulation within the tumor *in vivo* is based on the EPR effect. The liver metastasis generated by an intrasplenic injection of T-HCT 116 cells was clearly visualized by the NIR-conjugated anti-HLA probe but not by the orange-red fluorescent signal derived from the tdTomato reporter. This result demonstrated the superiority of the NIR probes over the tdTomato reporter protein at enhancing tissue penetration. In another xenograft model, patient-derived xenografts (PDX) of LC11-JCK (human non-small cell lung cancer) were successfully visualized using the NIR-conjugated macromolecule probe without any genetic modification. These results suggested that NIR-conjugated macromolecule, preferably, anti-HLA antibody probe is a valuable tool for the detection of human tumors in experimental metastasis models using whole-body imaging.

## Introduction

Human tumor xenograft (subcutaneous) models have been very popular *in vivo* models in oncology research. However, these models may not adequately reflect the pathophysiological environments in which cancer cells exist [1]. Liver metastasis xenograft models in relevant orthotopic locations, such as colorectal tumors metastasized to the liver, have been developed by intrasplenic (*isp*) injection of tumor cells into immunodeficient mice [2]. We have previously developed a reliable model system for assaying hematogenous liver metastases of pancreatic and colorectal cancers in NOG mice [3], [4]. The efficacy of farnesyl transferase inhibitors (FTIs) against HCT 116 (colorectal cancer) cells was evaluated in this model, and the effectiveness of this treatment was demonstrated by the prolonged survival times of mice treated with FTIs [4]. In most cases, treatment effectiveness is assessed in terms of survival or gross findings in the liver, as animal sacrifice is usually required. Therefore, novel and less invasive approaches for preclinical studies are required to evaluate the effectiveness of anti-tumor drugs *in vivo*.

In particular, optical bioimaging without radioactive tracers or ionizing radiation is suitable for such preclinical studies and facilitates serial measurements of xenografts, even when located intraperitoneally or in other orthotopic locations [5]. However, optical probes labeled with fluorochromes that emit light in the 400­–700 nm range, such as green fluorescent protein (GFP), have limited tissue penetration and high tissue autofluorescence [6]. Hence, fluorescent proteins (FPs) that have much longer wavelengths than GFP have been developed; these proteins fluoresce as orange-red and far-red, avoiding absorption by hemoglobin at wavelengths below 600 nm [7], [8]. The major bottleneck in most bioimaging experiments using fluoroproteins is the requirement to have previously transfected the corresponding gene into the target cells.

It is known that macromolecules such as albumin, transferrin, immunoglobulin, and α2-macroglobulin accumulate in solid tumors through the enhanced permeability and retention (EPR) effect caused by leaky vasculature within the tumor [9], [10]. This effect can facilitate binding between receptors and ligands, such as antibodies and adhesion molecules. Recently, Keereweer et al. reported the detection of oral cancer in an orthotopic mouse model using near-infrared (NIR) fluorescence agents that targeted either the αvβ3 integrins or the EPR effect [11]. In mice xenograft models, human and mouse cells display different major histocompatibility complex (MHC) surface antigens [12]. Therefore, these antigens are good target molecules for distinguishing between human cells and recipient mouse cells in xenograft tissue sections [13], [14]. Because of this, antibodies against the MHC class I antigens (HLA-A, -B, and -C in humans) such as the anti-HLA-ABC antibody, have been used as “Xenograft markers” for the detection of human cells by flow cytometry, immunoblotting, and immunohistochemical staining. Currently, there are no reports describing the use of this anti-HLA antibody as an *in vivo* imaging probe. Therefore, we sought to develop a versatile method using anti-HLA antibody for the detection of human tumors *in vivo* without the need for fluoroprotein expression. The anti-HLA-ABC antibody was conjugated with molecules that fluoresce in the NIR optical spectrum (650–900 nm), reducing background fluorescence and enhancing tissue penetration compared with fluorescent probes of shorter wavelengths. We assessed the feasibility of tumor detection in various xenotransplantation models using an NIR-conjugated anti-HLA antibody that targeted either the EPR effect or antigen–antibody binding. We showed that the NIR-probe was superior to the tdTomato reporter protein at enhancing tissue penetration *in vivo*. These results suggested that NIR-conjugated anti-HLA antibody probe is a valuable tool for the detection of human tumor xenografts in experimental mouse models using whole-body optical imaging.

## Materials and Methods

### Cell Culture

The human colorectal cancer cell line HCT 116 and the human pancreatic cancer cell line BxPC-3 were obtained from the American Type Culture Collection (Manassas, VA, USA) and were maintained in McCoy’s 5A and RPMI-1640 medium (Sigma, St. Louis, MO, USA), respectively, supplemented with antibiotics and 10% fetal bovine serum. Cells were incubated in a humidified incubator (37°C, 5% CO_2_) and were passaged upon reaching 80% confluence. To establish HCT 116 cell expressing the orange-red fluorescent protein tdTomato (Abs/Em = 554/581 nm) (T-HCT 116) as a control for fluoroimaging, HCT 116 cells were transfected with the ptdTomato-N1 vector (Clontech Laboratories, Inc. Mountain View, CA, USA) using magnetofection (Oz Biosciences, France) according to the manufacturer’s instructions. Two days after transfection, 500 µg/ml of neomycin (Invitrogen Corp., Carlsbad, CA) was added, and the cultures were maintained until cell death ceased.

### NIR fluorescent agents

For the direct detection of human tumors *in vivo*, the near-infrared (NIR)-conjugated anti-HLA antibody (NIR-αHLA) was prepared as follows. The mouse monoclonal anti-human HLA-ABC antibody clone W6/32 (IgG_2a_; Cedarlane Laboratories USA Inc., Burlington, NC, USA) and an isotype-matched mouse IgG_2a_ antibody (SouthernBiotech, Birmingham, AL, USA) were conjugated to the IVIS XenoLight^TM^ CF770 (Abs/Em = 770/797 nm) fluorochrome using the Fluorescent Dye kit for *In Vivo* Imaging (Caliper Life Sciences, Hopkinton, MA, USA) according to the manufacturer’s instructions. The absorbance of the NIR-conjugated antibodies was measured at 280 and 770 nm using a SmartSpec™ 3000 spectrophotometer (BioRad Laboratories, Hercules, CA, USA). The final concentration of the antibody conjugate and the degree of labeling (DOL) were calculated using the following formulae:



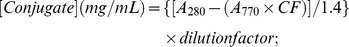



CF is the absorbance correction factor (0.06 for XenoLight CF770), and the value 1.4 is the extinction coefficient of whole (H+L) IgG.



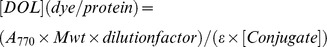



Mwt is the molecular weight (150,000 for IgG), and ε is the molar extinction coefficient (220,000 for XenoLight CF770). Bovine serum albumin (BSA; Nacalai, Kyoto, Japan) was also conjugated to the XenoLight^TM^ CF770 fluorochrome (NIR-BSA), and the DOL was calculated using the extinction coefficient (0.66) and Mwt (67,000) of BSA. The DOL in the NIR-αHLA (0.89 mg protein/mL), the NIR-conjugated mouse isotype control IgG_2a_ immunoglobulin (NIR-Isotype; 0.60 mg protein/mL), and BSA (0.73 mg protein/mL) were 1.34, 1.42, and 0.72 dye/protein, respectively. Free fluorochrome (Free NIR) and fluorochrome-glycine (NIR-Glycine), which is produced when the conjugation procedure is quenched by the addition of excess glycine (Nacalai, Kyoto, Japan), were used as negative control probes.

### Animals

All mice studies were conducted in strict accordance with the Guide for the Care and Use of Laboratory Animals from the Central Institute for Experimental Animals. All experimental protocols were approved by the Animal Care Committee of the CIEA (Permit Number: 11029A). All surgeries were performed under isoflurane anesthesia, and all efforts were made to minimize animal suffering. For whole-body optical imaging, we established an immunodeficient hairless mouse strain, the BALB/cA *Rag2^null^ Il2rg^null^ nude* (C.Cg-*Rag2^tm1^Il2rg^tm1Sug^ Foxn1^nu^*/Jic; abridged name: BRG nude) strain. This strain was created by crossing the BALB/cA *Rag2^null^ Il2rg^null^* (C.Cg-*Rag2^tm1^Il2rg^tm1Sug^*/Jic; abridged name: BRG) strain [15] and the BALB/cA *nude* (C.Cg-*Foxn1^nu^*/Jic; abridged name: nude) strain [16]. To produce an orthotopic pancreatic cancer model, immunodeficient NOG (NOD.Cg-*Prkdc^scid^ Il2rg^tm1Sug^*/ShiJic) mice were used as transplantation hosts [17].

### Xenograft Models

To generate xenograft models and liver and hematogenous metastasis models, 1×10^5^ HCT 116 and T-HCT 116 cells were suspended in 0.1 mL of serum-free medium and then subcutaneously (*sc*) transplanted into the left flank of 7–9 week-old BRG nude mice (n = 10 and 8, respectively). To generate tumor xenografts in both the subcutaneous spaces of 7–9 week-old BRG nude mice (n = 5), 1×10^6^ HCT 116 and BxPC-3 cells were suspended in 0.1 mL of serum free medium and were *sc* transplanted into the left and right flank, respectively. Liver metastases of human colorectal cancer cells were generated by intrasplenic (*isp*) injections of 1×10^5^ T-HCT 116 cells and 1×10^6^ HCT 116 cells into BRG nude mice (n = 3 and 3, respectively), followed by splenectomy under isoflurane anesthesia [4]. Hematogenous metastases of HCT 116 cells were generated by an intravenous (*iv*) injection of 1×10^5^ cells into BRG nude mice (n = 3). To generate an orthotopic implantation model of pancreatic cancer, BxPC-3 cells (1×10^6^ cells/head) were injected intrasplenically (*isp*) into splenic vein-ligated 9 week-old NOG mice, and a splenectomy was then performed (n = 2). Human non-small cell lung cancer (NSCLC) xenografts [18] were created by the *sc* implantation of LC11-JCK cells by trocar cannula into the left flank of BRG nude mice (n = 4).

### In vivo animal imaging

Spectral fluorescence images were obtained using the Kodak *In Vivo* Imaging System FX (Carestream Health, Inc. Rochester, NY, USA) and the IVIS SpectrumCT (Caliper Life Sciences, Hopkinton, MA, USA). After an intravenous injection with 100 µL of the NIR fluorochrome-conjugated probes, whole-body fluorescence images were obtained under isoflurane anesthesia. The NIR-conjugated macromolecule probes (including NIR-BSA, NIR-Isotype, and NIR-αHLA) were detected at wavelengths of 720 nm (excitation) and 790 nm (emission); the tdTomato fluoroprotein was detected at an excitation wavelength of 535 nm and an emission wavelength of 600 nm using the Kodak *In-Vivo* Imaging System FX. The NIR fluorescent signal was detected at a 745 nm excitation wavelength and an 800 nm emission wavelength using the IVIS SpectrumCT. Bright-field photographs were obtained for each imaging time. The merged bright-field photographs and fluorescence images were generated using the Kodak Molecular Imaging software SE5.0 (Carestream Health, Inc.) and the Living Image software 4.1.3 (Caliper Life Sciences). Fluorescent intensity was quantified in the region of interest (ROI). Identical illumination settings (lamp voltage, filters, f/stop, field of views, binning) were used for acquiring all images, and the fluorescence emission was normalized to photons per second per centimeter square per steradian (p/s/cm^2^/sr) in the quantitative analysis. All NIR fluorescent images were acquired using 1 second-exposure time (f/stop = 2) and displayed in the same scale of fluorescent intensity. Mice were sacrificed by exsanguination under isoflurane anesthesia immediately after the completion of the imaging. Abdominal surgery was then conducted to clearly show the cancer cell engraftments and to enable *in situ* and *ex vivo* optical imaging using the same system.

### Immunohistochemical staining

Mice were euthanized by exsanguination under anesthesia, and xenograft tumors were excised and embedded in OCT compound (Sakura Finetek Japan Co., Ltd., Tokyo, Japan) and frozen in liquid nitrogen. Five-micron-thick serial frozen sections were prepared and fixed with 4% (v/v) paraformaldehyde (Wako Pure Chemical Industries, Ltd., Osaka, Japan). Nonspecific peroxidase activity was quenched by incubation with 0.3% hydrogen peroxide for 5 min. Sections were incubated primarily with rabbit polyclonal anti-red fluorescent protein (RFP; Abcam, Cambridge, UK), goat polyclonal mouse anti-IgG_2a_ (Bethyl Laboratories, Montgomery, TX, USA) antibodies, and rat monoclonal mouse anti-CD31 (PECAM-1) (Dianova, Hamburg, Germany) antibodies for overnight at 4°C. Signals were detected using a immune-enzyme polymer method (Nichirei, Tokyo, Japan) using a 3,3′-diaminobenzidine tetrahydrochloride (DAB; Dojindo Laboratories, Kumamoto, Japan) substrate as a chromogen. Sections were counterstained with hematoxylin.

### Statistical Analyses

Statistical analyses were performed with the Prism 5 software (GraphPad Software, CA, USA).

## Results

### Confirmation of NIR-αHLA specificity for human cancer cells

**Figure 1:**
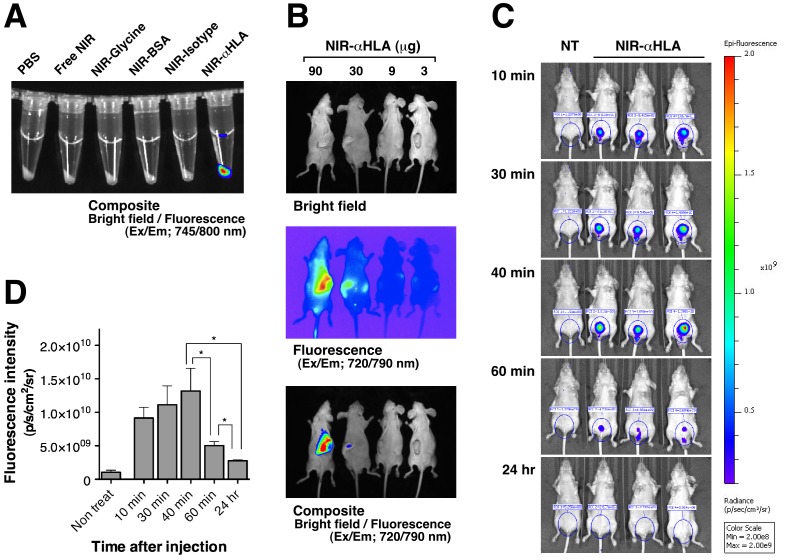
Reactivity of the NIR-αHLA probe with HCT 116 (human colorectal cancer) cells *in vitro* and *in vivo*. (A) HCT 116 cells were incubated with various NIR-probes, and the *in vitro* fluorescence signals were specifically detected at wavelengths of 745/800 nm, which were overlaid onto a bright-field image. (B) *In vivo* fluorescence images of HCT 116 tumor-bearing BRG nude mice. Dose-related effects of the NIR fluorescence intensities after *iv* injection of various amounts of NIR-αHLA probe can been observed. Fluorescent signal from the NIR-αHLA probe was specifically detected at wavelengths of 720/790 nm. The bright-field image is shown in the top panel, the fluorescent image is shown in the middle panel, and the overlay image is shown in the bottom panel. (C) The time course of the NIR fluorescence intensity of BRG nude mice that had received an *iv* injection of the NIR-αHLA probe. (D) Fluorescence intensities were quantified using ROIs of equivalent-sized areas from the lower abdominal regions at the indicated time points. Data are presented as the mean ± SD of three individual mice (Student’s *t* test, ^*^ *p* value of 40 min and 60 min = 0.0390, for 40 min and 24 hr = 0.0151, and for 60 min and 24 hr = 0.0313).

To confirm the specificity of the fluorochrome-conjugated probes *in vitro*, HCT 116 (human colorectal cancer) cells were treated with Free NIR, NIR-Glycine, NIR-BSA, NIR-Isotype (isotype control), or NIR-αHLA. Fluorescence imaging of the NIR-probes was then conducted using the excitation/emission 745/800 nm filter sets. Only the cells treated with NIR-αHLA fluoresced, and fluorescence were not observed in the cells treated with the other NIR-probes (Figure 1A).

To assess whether the NIR-αHLA probe could be used to visualize human tumors *in vivo*, BRG nude mice were *sc* transplanted with HCT 116 cells and were imaged after *iv* injection with different amounts of the NIR-αHLA probe. The mice were injected with the NIR-αHLA probe (90, 30, 9, or 3 µg/mouse) and were imaged on day 1 (Figure 1B). The NIR signal was observed in the tumor regions of the mice that had received an injection of more than 30 µg NIR-αHLA probe. The rapid clearance of the NIR-αHLA probe was confirmed by fluorescence imaging (Figure 1C). The accumulation of fluorescence in the bladder peaked at 40 min, and the NIR fluorescence disappeared within 24 hr after NIR-αHLA probe injection (Figure 1D).

### Specificity of NIR-conjugated macromolecule probe accumulation in tumor xenografts

**Figure 2:**
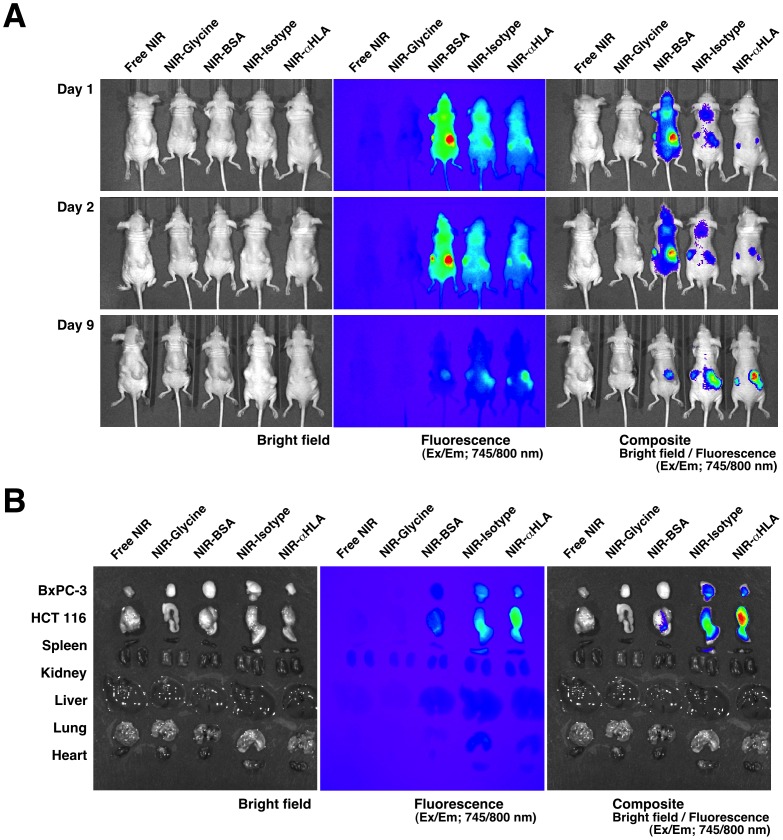
Proof of concept experiments for the *in vivo* imaging of human tumors with the NIR-probes. (A) *In vivo* fluorescence images of BxPC-3 and HCT 116 tumor-bearing BRG nude mice (left and right flank, respectively) were taken 1, 2, and 9 days after *iv* injections with various NIR-probes. (B) *Ex vivo* fluorescence images of the BxPC-3 and HCT 116 xenografts and tissues of recipient BRG nude mice. The bright-field photograph is shown in the left panel, the fluorescence image is shown in the center panel, and the overlay image is shown in the right panel. The fluorescent signal from the NIR-probes were specifically detected at 745/800 nm using an IVIS SpectrumCT.

As seen in an *in vitro* study of HCT 116 cells, fluorescence was observed in BxPC-3 (human pancreatic cancer) cells *in vitro* only when the cells were treated with the NIR-αHLA probe (data not shown). To validate the NIR-αHLA probe specificity for tumor cells *in vivo*, BRG nude mice were *sc* transplanted with BxPC-3 cells and HCT 116 cells on the left and right flanks, respectively, and were imaged after *iv* injection of the different NIR-probes with equivalent amounts of fluorochrome. The mice were imaged on days 1, 2, and 9 after NIR-probe injection (Figure 2A). On day 1 after probe injection, the mice injected with NIR-BSA, NIR-Isotype, or the NIR-αHLA probe accumulated fluorescent signals in the tumor regions. However, the mice injected with the small molecular probes (Free NIR and NIR-Glycine) did not show any fluorescent signal accumulation. These results implied that the NIR-conjugated macromolecules (BSA and immunoglobulin (IgG)) accumulated in the tumors mainly as a result of the EPR effect. Nine days after NIR-probe injection, the fluorescent signals in the tumor regions were decreased in the mice that had been injected with NIR-BSA and NIR-Isotype; overall, a decrease in background fluorescence was also observed. In contrast, the fluorescence signals derived from the NIR-αHLA probe seemed to be retained in the tumor region despite the disappearance of the background fluorescence. This specificity and prolonged retention of the NIR-αHLA probe coincided with the *ex vivo* imaging (Figure 2B). The strongest NIR signals were observed in the tumors excised from mice that had been injected with the NIR-αHLA probe.

**Figure 3:**
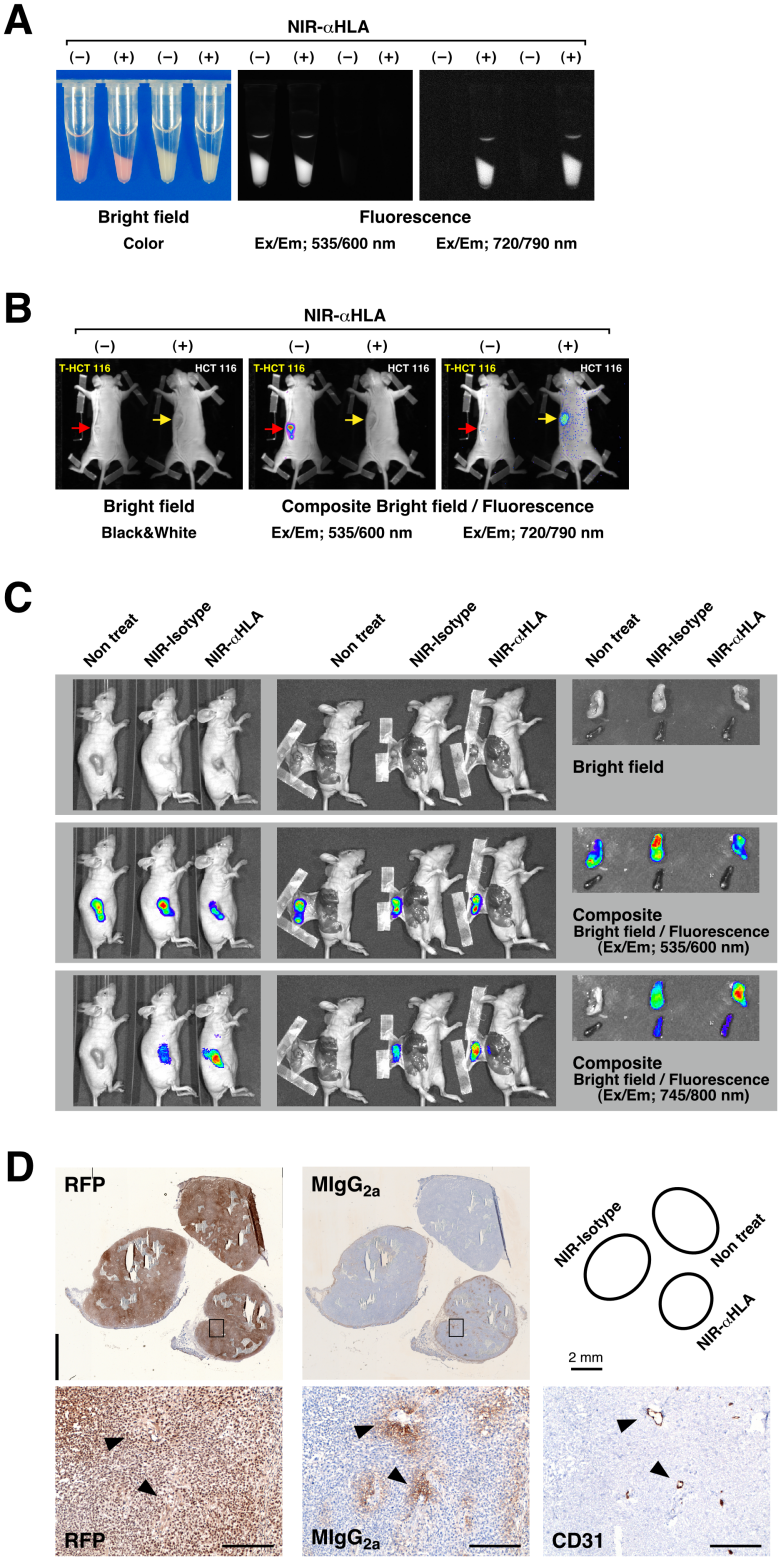
Validation of *in vivo* imaging of human tumors with the NIR-conjugated macromolecule probes. (A) Bright-field images and fluorescence images of the T-HCT 116 cells (which express tdTomato) and HCT 116 cells *in vitro*. Fluorescent signal from the orange-red fluorescent protein tdTomato and the NIR-αHLA probe were specifically detected at wavelengths of 535/600 nm and 720/790 nm, respectively. The absence or presence of the NIR-αHLA antibody is indicated as NIR-αHLA (–) or (+), respectively. (B) *In vivo* fluorescence images of T-HCT 116 and HCT 116 tumor-bearing BRG nude mice. The NIR fluorescence intensity 2 days after *iv* injection of the NIR-αHLA probe can be observed. Fluorescent signal from tdTomato and NIR-αHLA probe were specifically detected at wavelengths of 535/600 nm and 720/790 nm, respectively, using the Kodak *In-Vivo* Imaging System FX. The absence or presence of the NIR-αHLA probe is indicated as NIR-αHLA (–) or (+), respectively. The red and yellow arrows indicate engraftment sites of T-HCT 116 cells and HCT 116 cells, respectively. (C) Fluorescent signal of the NIR-conjugated macromolecule probes co-localized with tdTomato in T-HCT 116 cells in tumor-bearing BRG mice. The fluorescent signals at 535/600 nm and 745/800 nm were overlaid (composite) using Living Image software 4.1.3. Li; liver, Sp; spleen. (D) Immunohistochemical staining of dissected tumors; anti-RFP (RFP; left), anti-mouse IgG_2a_ (MIgG_2a_; center), and anti-CD31 (CD31; right); Enlarged view of boxed area shown below. Arrowheads indicate same position the on serial section. Scale bar, 200 µm.

To confirm the specificity of the NIR-conjugated macromolecule probe *in vitro*, orange-red fluorescent protein tdTomato-expressing HCT 116 cells (T-HCT 116 cells) and HCT 116 cells were treated with the NIR-αHLA probe (Figure 3A). Fluorescence imaging of tdTomato and the NIR-αHLA probe was then conducted using the excitation/emission filter sets of 535/600 and 720/790 nm, respectively. The cells treated with the NIR-αHLA probe showed fluorescent signals with the 720/790 nm filter set; however, the fluorescent signals were not observed in the cells in the absence of the NIR-αHLA probe. Fluorescence of the accumulated tdTomato protein was detected only in T-HCT 116 cells, using the 535/600 nm filter set. When transplanted cancer cells became visible and palpable, the mice were injected with the NIR-αHLA probe (90 µg/mouse). The mice were imaged 2 days after NIR-αHLA probe injection (Figure 3B). Fluorescence of the NIR signal was observed in the tumor regions within 1 day of probe injection, was optimal at 2 days, and remained visible for up to 14 days after antibody injection (data not shown). The T-HCT 116 cells were used as a fluorescence imaging control. Fluorescence of the accumulated tdTomato protein was detected using the 535/600 nm filter set, but the fluorescent signals were not detected with the 720/790 nm filter set. These results indicated that this optical imaging system could detect both fluoroprobes simultaneously with no cross-interference. Figure 3C shows a series of whole-body (left panels), dermabrasion (center panels), and *ex vivo* (right panels) fluorescence images that were obtained 24 hr after the administration of the NIR-Isotype or NIR-αHLA probes (90 µg/mouse). The specificity of the NIR-conjugated macromolecule probe for human tumors was demonstrated by the co-localization of the NIR and orange-red fluorescence signals within engrafted T-HCT 116 cells *in vivo* and *ex vivo*. The specific accumulation of the NIR-conjugated macromolecule probes within the T-HCT 116 xenograft was confirmed by immunohistochemical staining with anti-RFP antibody and anti-mouse immunoglobulin antibody. The injected NIR-conjugated macromolecule probes (mouse immunoglobulin G_2a_) were localized around blood vessels, which were stained by the specific endothelial marker CD31 (Figure 3D).

**Figure 4:**
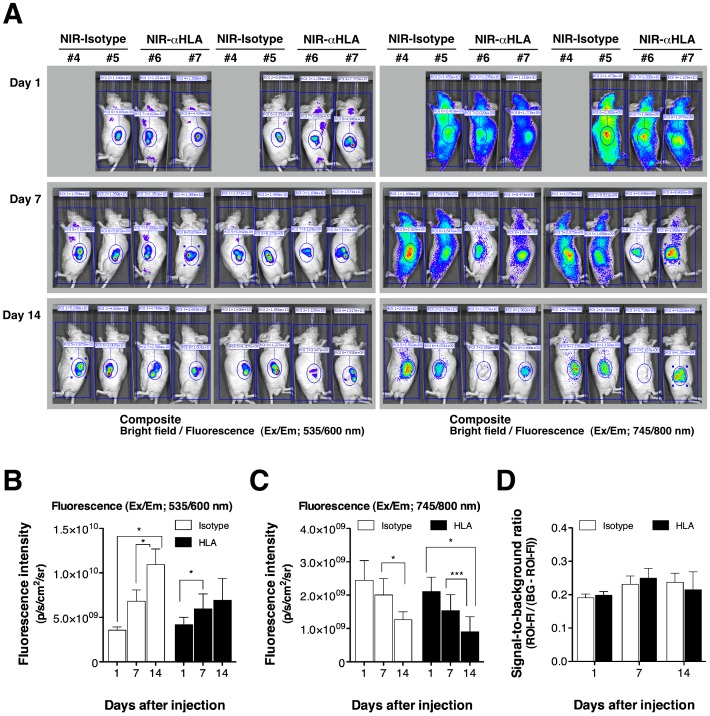
Time course of fluorescence intensities in human tumor xenografts after *iv* injection of NIR-conjugated macromolecule probes. (A) The fluorescent signal from the tdTomato (left panels) and NIR-probes (right panels) in subcutaneous T-HCT 116 tumor-bearing BRG nude mice (left and right flank, respectively) were detected at 1, 7, and 14 days after *iv* injection with NIR-probes, at wavelengths of 535/600 nm and 745/800 nm, respectively, using an IVIS SpectrumCT. (B) Fluorescence intensities of the tdTomato signal were quantified using ROIs of equivalent-sized areas from the tumor regions at the indicated time points (Student’s *t* test, NIR-Isotype probe injected: ^*^ *p* value for 1 day and 14 days = 0.0216 and for 7 days and 14 days = 0.0126; NIR-αHLA probe injected: ^*^ *p* value for 1 day and 7 days = 0.0299). (C) Fluorescence intensities of the NIR-probe signal were quantified using ROIs of equivalent-sized areas from the tumor regions at the indicated time points (Student’s *t* test, NIR-Isotype probe injected: ^*^ *p* value for 7 days and 14 days = 0.0176; NIR-αHLA probe injected: ^*^ *p* value for 1 day and 14 days = 0.0172, and ^***^ *p* value for 7 days and 14 days<0.0001). Data were presented as the mean ± SD of four individual tumors (two individual tumors in case of day1-#4 mouse. (D) The signal-to-background ratio (S/B) at 1, 7, and 14 days post-administration of the NIR-probes was calculated using the following formula: S/B  =  (fluorescence intensity of ROI)/((background intensity) – (fluorescence intensity of ROI)). In each case, the background was derived from equivalently sized areas containing the same number of pixels.

The human tumors engrafted in BRG nude mice were successfully visualized by the NIR-Isotype probe and NIR-αHLA probes. To compare the NIR-Isotype and NIR-αHLA probes in terms of their ability to detect human tumor cells *in vivo*, the ROIs of that the fluorescence intensities of T-HCT 116 tumor and of background signal were quantified at different time points after probe administration (Figure 4). In contrast to the increasing orange-red fluorescent signals in T-HCT 116 cells (Figure 4B), both NIR fluorescent signals decreased over time (Figure 4C). The fluorescence intensity ratio between the tumor and background for NIR-probes is defined as the signal-to-background (S/B) ratio. The S/B ratio of NIR-Isotype and NIR-αHLA did not show significant differences between time points (Figure 4D). This result indicates that fluorescence imaging with the NIR-conjugated αHLA antibody was mainly based on the EPR effect.

### NIR-conjugated macromolecule probes facilitate visualization of human tumors in various transplantation models

**Figure 5:**
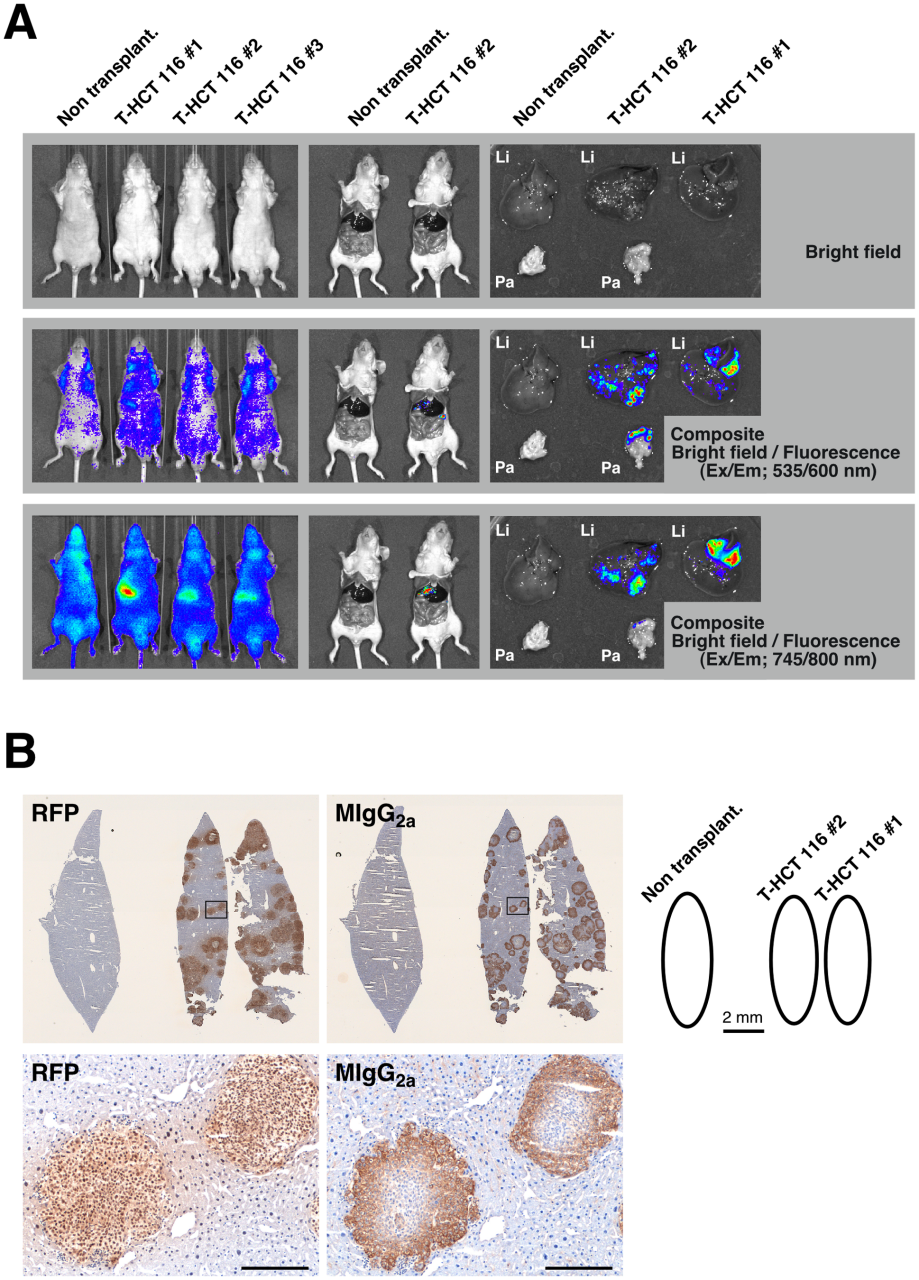
*In vivo* imaging of the liver metastasis model with the NIR-αHLA probe. (A) *In vivo* fluorescence images of T-HCT 116 tumor-bearing BRG nude mice. The NIR fluorescence intensity 24 hr after *iv* injection of the NIR-αHLA probe can be observed using an IVIS SpectrumCT. The fluorescent signal from tdTomato and the NIR-αHLA probe were specifically detected at wavelengths of 535/600 nm and 745/800 nm, respectively. Nontransplant indicates that the BRG mouse had not received the T-HCT 116 cell transplant. Li; liver, Pa; pancreas. (B) Immunohistochemical staining of dissected livers; anti-RFP (RFP; left), and anti-mouse IgG_2a_ (MIgG_2a_; right); Enlarged view of boxed area shown below. Scale bar, 200 µm.

Following these initial proof of principle experiments, the NIR-conjugated macromolecule probes were used as imaging probes in subsequent experiments using various types of xenotransplantation models in BRG nude mice. We first attempted to detect the liver metastasis of colorectal cancer generated by the *isp* injection of T-HCT 116 cells. Three weeks after transplantation, the mice were treated with the NIR-αHLA probe (90 µg/mouse). Figure 5A shows a series of whole-body (left panels) and laparotomized body (center panels) fluorescence images from mice inoculated with T-HCT 116 cells, as well as *ex vivo* (right panels) fluorescence images. These pre- and post-mortem images were obtained 24 hr after administration of the NIR-αHLA probe. The fluorescence intensities observed in the mouse livers were higher than those in the rest of the body using fluorescence imaging with the 745/800 nm filter set in all T-HCT 116 cell–transplanted mice (3 out of 3). By contrast, we did not visualize the orange-red fluorescent signal of the liver-metastasized T-HCT 116 cells with the 535/600 nm filter set. *Ex vivo* imaging with the NIR-αHLA probe also demonstrated a clear demarcation of the tumor from the surrounding healthy liver tissue (Figure 5A, bottom panels). Furthermore, the NIR fluorescent signals were coincident with the orange-red fluorescent signal of the T-HCT 116 cells in *ex vivo* imaging. This specific accumulation of the NIR-αHLA probe in the liver-metastasized T-HCT 116 xenografts was confirmed by immunohistochemical staining with anti-RFP antibody and anti-mouse IgG_2a_ antibody (Figure 5B). The NIR-αHLA probes, which were detected by anti-mouse IgG_2a_ antibody, were localized within T-HCT 116 xenograft tumors, which were stained with anti-RFP antibody.

**Figure 6:**
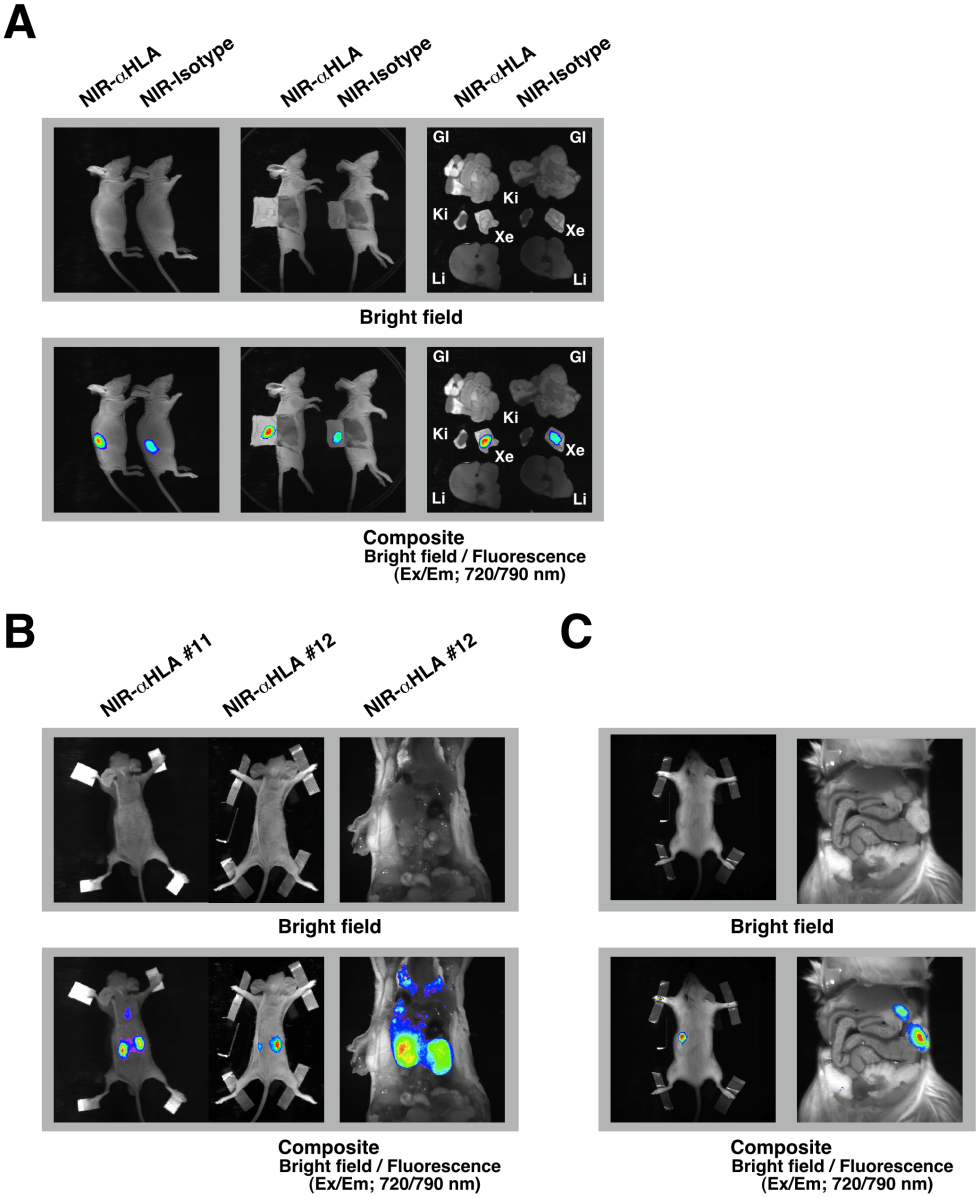
*In vivo* imaging with the NIR-αHLA probe detected human tumors in various transplantation models. (A) *In vivo* fluorescence images of BRG nude mice that had been implanted with small pieces of LC11-JCK xenograft tumors were taken 48 hr after *iv* injection with the NIR-αHLA probe or the NIR-Isotype probe (left panels). In laparotomized body *ex vivo* fluorescence images (center panels) and *ex vivo* fluorescence images (right panels), the fluorescent signal was specifically detected at wavelengths of 720/790 nm using the Kodak *In-Vivo* Imaging System FX. GI; gastrointestinal tract, Ki; kidney, Xe; LC11-JCK tumor xenograft, Li; liver. (B) *In vivo* fluorescence images of BRG nude mice that had received an intravenous (via tail-vein) injection of 1×10^5^ HCT 116 cells were taken 48 hr after *iv* injection with the NIR-αHLA probe (left panels). Laparotomized body *ex vivo* fluorescence images (right panels) are also shown. A bright-field image is shown in the top panel, a fluorescence image is shown in the center panel, and the composite image is shown in the right panel. (C) An *in vivo* fluorescence image of a NOG mouse that had received an intrasplenic injection of 1×10^6^ BxPC-3 cells was taken 48 hr after *iv* injection with the NIR-αHLA probe (left panels). Laparotomized body *ex vivo* fluorescence images (right panels) are also shown. The fluorescent signal from the NIR-probes was acquired using the Kodak *In-Vivo* Imaging System FX.

In subsequent experiments, we attempted to detect LC11-JCK xenograft tumors, which were established from human surgical specimens by serial passage in the subcutaneous spaces of immunodeficient BALB/cA nude mice. Recently, this type of tumor resource has been called a “Patient-Derived tumor Xenograft (PDX) model” [19]. To apply our versatile technique for *in vivo* imaging to the PDX model, 9 pieces of a 1-mm cubically dissected LC11-JCK xenograft were implanted into the subcutaneous spaces of BRG nude mice. After 4 weeks, the mice were treated with the NIR-αHLA probe or NIR-Isotype probes. Figure 6A shows a series of whole-body images that were obtained 2 days after administration of the NIR-probes. A bright-field image is shown in the top panel. When the fluorescent signals are overlaid onto the bright-field image, *in vivo* maintenance of the xenograft tumors can be visualized using both the NIR-αHLA and the NIR-Isotype probes. Dermabrasion and *ex vivo* fluorescence imaging with the NIR-conjugated macromolecule probes confirmed specific detection of the LC11-JCK tumors in whole-body imaging of the PDX model.

In the same manner as in the PDX model, we attempted to detect non-genetically modified HCT 116 cells by using NIR-conjugated macromolecule probes in various types of xenotransplantation models. We attempted to detect the unpredictable metastases of HCT 116 cells following *iv* injection of the cells. Four weeks after inoculation, the mice were treated with the NIR-αHLA probe (90 µg/mouse). Figure 6B shows a series of whole-body (left panels) and *ex vivo* laparotomized body (right panels) fluorescence images of mice inoculated with HCT 116 cells. These pre- and post-mortem images were obtained 2 days after administration of the NIR-αHLA probe. In the hematogenous metastasis model, following *iv* inoculation with HCT 116 cells, the development of renal metastases was detected noninvasively using this method. An orthotopic model of pancreatic cancer was prepared through a modified intrasplenic transplantation of BxPC-3 cells. Four weeks after transplantation, the mice were treated with the NIR-αHLA probe. Figure 6C shows a series of whole-body (left panels) and *ex vivo* laparotomized body (right panels) fluorescence images from mice inoculated with 1×10^6^ BxPC-3 cells. Pre- and post-mortem images were obtained 2 days after the administration of NIR-αHLA probe (90 µg/mouse). In the whole-body imaging experiments, fluorescent signals were observed in areas similar to those observed in the renal metastasis model. However, the *ex vivo* laparotomized body fluorescence imaging revealed that the NIR fluorescent signals were located in the pancreas.

## Discussion

This study demonstrates that whole-body optical imaging using NIR-conjugated macromolecule probes can detect human tumors in immunodeficient mice.

*In vivo* analyses of the growth and metastases of xenografts are crucial to the evaluation of new anti-cancer drugs and to the identification of molecules that play key roles in tumor malignancy. In experiments using subcutaneous xenograft models, measuring tumor volume can offer a clear evaluation of drug efficacy. In contrast, although orthotopic implantations and hematogenous metastasis models are more accurate [20], drug efficacy, particularly the tumor-suppressing effect of the drug, is evaluated only by autopsy or overall survival. These methods are invasive and time-consuming. In 2000, Yang et al. published a groundbreaking report on whole-body optical imaging using green fluorescent protein (GFP)-expressing cancer cells [21]. This work allowed unprecedented continuous, noninvasive visual assessments of malignant growth and spread in live animals. Tracking the GFP-expressing cancer cells *in vivo* is far more sensitive and rapid than cumbersome histological or immunohistochemical procedures [22]. Recently, the use of near-infrared (NIR) wavelengths (700–900 nm) instead of visible wavelengths (400–700 nm) was found to be advantageous for *in vivo* imaging, owing to the very low autofluorescence of tissue and the high level of tissue penetration of these wavelengths [23], [24]. Although, we failed to visualize liver metastasis of tdTomato-expressing HCT 116 human colorectal cancer (T-HCT-116) cells by orange-red fluorescent protein tdTomato with the 535/600 nm filter set, the fluorescent signal from liver-metastasized T-HCT 116 cells was clearly detected by both NIR-αHLA (Figure 5A) and NIR-Isotype (data not shown) probes with the 745/800 nm filter set. This result confirmed that the NIR wavelength is superior to the orange-red fluorescence wavelength in terms of tissue penetration. To overcome the disadvantage of using tdTomato fluoroprotein, the spectra of the fluorescent proteins should be shifted to longer wavelengths. The infrared fluorescent protein alternative to GFP was originally isolated from *D. radiodurans* and showed superior utility in whole-body imaging of internal mammalian tissues [25], [26]. However, the issue of which fluorescent protein expressing cell lines need to be established remains.

Solid tumors characteristically exhibit an accumulation of macromolecules, as a result of the EPR effect, which arises from the leaky vasculature within the tumors [9], [10]. In this study, we examined the uptake of NIR-probes of different molecular sizes by conjugating the NIR fluorochrome to glycine (75 Da), BSA (67 kDa), and immunoglobulins (150 kDa). The NIR-conjugated macromolecules, including NIR-BSA, NIR-Isotype, and NIR-αHLA probe, accumulated in both BxPC-3 and HCT 116 xenograft tumors; in contrast, small molecular NIR-probes, such as NIR-glycine and free NIR fluorochrome, could not be retained in either the BxPC-3 or HCT 116 xenograft tumors (Figure 2). These results indicate that the principal mechanism underlying optical imaging with the NIR-conjugated macromolecule probes may be due to the EPR effect. Immunohistochemical staining with anti-mouse immunoglobulin G_2a_ revealed the localization of NIR-αHLA probes that had leaked from blood vessels. This localization supports the idea that the EPR effect, caused by leaky vasculature within the tumor, is the underlying mechanism [9], [10].

In the subcutaneous transplantation model, all T-HCT 116 xenografts expressing orange-red fluorescence protein were successfully detected at both the Ex/Em 535/600 nm (tdTomato fluorescence reporter) and Ex/Em 745/800 nm (NIR-probe fluorescence) wavelengths (6 out of 6). In addition, all visible and palpable BxPC-3 and HCT 116 xenografts were also detected by NIR-probes (3/3 and 5/5, respectively). In a liver metastasis model with T-HCT 116 reporter cells, whole-body imaging with the 535/600 nm wavelength filter set failed to visualize liver-metastasized T-HCT 116 cells (0 out of 3). In contrast, the BRG nude mice that received *isp* transplantation with T-HCT 116 reporter cells emitted sufficient NIR fluorescent signal from the abdomen in whole-body imaging (3 out of 3). *Ex vivo* imaging of the liver metastasis model clearly demarcated tumors from the surrounding liver cells during detection of the fluorescent signal with both Ex/Em 535/600 nm and 745/800 nm wavelengths.

Using *ex vivo* imaging, but not whole-body imaging, we identified a mouse in which the intrasplenically transplanted T-HCT 116 cells had metastasized to the pancreas. Generally, it is known that the EPR-effect can be observed in almost all human cancers with the exception of hypovascular tumors such as prostate cancer or pancreatic cancer [27]. This characteristic might explain the unsuccessful detection of an NIR fluorescent signal in the tumor xenografts formed in the pancreas. Alternatively, the NIR fluorescent signal in xenograft tumors formed in the pancreas might be masked by the strong NIR fluorescent signal of liver-metastasized tumors. This hypothesis is supported by the successful whole-body imaging, using the NIR-αHLA probe, of the human pancreatic cancer (BxPC-3) xenograft formed in the pancreas(Figure 6C).

In human tumor xenografts and human tissue or cell transplantation models, anti-human HLA antibodies are powerful tools for detecting and distinguishing human cells from recipient mouse cells using immunohistochemical staining of tissue sections [13], [14]. In this study, we confirmed that *in vitro*, the human cancer cell lines HCT 116 and BxPC-3 were specifically detected by NIR-conjugated anti-HLA antibody (NIR-αHLA probe) but not by NIR-conjugated isotype-matched immunoglobulin (NIR-Isotype probe). However, both NIR-probes had a similar capacity to detect the HCT 116 and BxPC-3 human cancer cells *in vivo*. This result indicates that fluorescence imaging with NIR-conjugated anti-HLA antibody *in vivo* is mainly based on the EPR effect rather than antigen–antibody binding. The BRG nude strain, which lacks immunoglobulin [28], might have an EPR effect-prone internal environment. It is known that some cancer cells have lost the surface expression of HLA molecules. However, the complete loss of HLA class I molecules was found to occur in only 9% (6 of 70) of a group of esophageal squamous cell carcinoma (ESCC) patients and was not observed (0 of 34) in a group of head and neck squamous cell carcinoma (HNSCC) patients [29], [30]. Fortunately, our versatile method, which does not require any genetic modification of the target cancer cells, allows us to detect tumor xenografts by using BRG nude mice as recipients, even for cancers that have lost the surface expression of HLA class I antigen.

In this report, we demonstrated a simple method for detecting human xenograft tumors in immunodeficient mice: using NIR-conjugated macromolecule (immunoglobulin) probes. This versatile method for the *in vivo* imaging of human tumor xenografts should facilitate studies of cancer growth and metastasis and accelerate the development of potential chemotherapeutic agents.

## References

[1] Chang CJ, Tai KF, Roffler S, Hwang LH (2004) The immunization site of cytokine-secreting tumor cell vaccines influences the trafficking of tumor-specific T lymphocytes and antitumor efficacy against regional tumors. J Immunol 173: 6025–6032. 15528337 10.4049/jimmunol.173.10.6025

[2] Giavazzi R, Campbell DE, Jessup JM, Cleary K, Fidler IJ (1986) Metastatic behavior of tumor cells isolated from primary and metastatic human colorectal carcinomas implanted into different sites in nude mice. Cancer Res 46: 1928–1933. 3948174

[3] Suemizu H, Monnai M, Ohnishi Y, Ito M, Tamaoki N, et al (2007) Identification of a key molecular regulator of liver metastasis in human pancreatic carcinoma using a novel quantitative model of metastasis in NOD/SCID/gammacnull (NOG) mice. Int J Oncol 31: 741–751. 17786304

[4] Hamada K, Monnai M, Kawai K, Nishime C, Kito C, et al (2008) Liver metastasis models of colon cancer for evaluation of drug efficacy using NOD/Shi-scid IL2Rgammanull (NOG) mice. Int J Oncol 32: 153–159. 18097554

[5] Bibby MC (2004) Orthotopic models of cancer for preclinical drug evaluation: advantages and disadvantages. Eur J Cancer 40: 852–857. 15120041 10.1016/j.ejca.2003.11.021

[6] Troy T, Jekic-McMullen D, Sambucetti L, Rice B (2004) Quantitative comparison of the sensitivity of detection of fluorescent and bioluminescent reporters in animal models. Mol Imaging 3: 9–23. 15142408 10.1162/15353500200403196

[7] Deliolanis NC, Kasmieh R, Wurdinger T, Tannous BA, Shah K, et al (2008) Performance of the red-shifted fluorescent proteins in deep-tissue molecular imaging applications. J Biomed Opt 13: 044008. 19021336 10.1117/1.2967184 PMC2749214

[8] Lin MZ, McKeown MR, Ng HL, Aguilera TA, Shaner NC, et al (2009) Autofluorescent proteins with excitation in the optical window for intravital imaging in mammals. Chem Biol 16: 1169–1179. 19942140 10.1016/j.chembiol.2009.10.009 PMC2814181

[9] Matsumura Y, Maeda H (1986) A new concept for macromolecular therapeutics in cancer chemotherapy: mechanism of tumoritropic accumulation of proteins and the antitumor agent smancs. Cancer Res 46: 6387–6392. 2946403

[10] Greish K, Fang J, Inutsuka T, Nagamitsu A, Maeda H (2003) Macromolecular therapeutics: advantages and prospects with special emphasis on solid tumour targeting. Clin Pharmacokinet 42: 1089–1105. 10.2165/00003088-200342130-00002 14531722

[11] Keereweer S, Mol IM, Kerrebijn JD, Van Driel PB, Xie B, et al (2012) Targeting integrins and enhanced permeability and retention (EPR) effect for optical imaging of oral cancer. J Surg Oncol 105: 714–718. 21952950 10.1002/jso.22102

[12] Concha A, Esteban F, Cabrera T, Ruiz-Cabello F, Garrido F (1991) Tumor aggressiveness and MHC class I and II antigens in laryngeal and breast cancer. Semin Cancer Biol 2: 47–54. 1912518

[13] Machida K, Suemizu H, Kawai K, Ishikawa T, Sawada R, et al (2009) Higher susceptibility of NOG mice to xenotransplanted tumors. J Toxicol Sci 34: 123–127. 19182442 10.2131/jts.34.123

[14] Hasegawa M, Kawai K, Mitsui T, Taniguchi K, Monnai M, et al (2011) The reconstituted 'humanized liver' in TK-NOG mice is mature and functional. Biochem Biophys Res Commun 405: 405–410. 21238430 10.1016/j.bbrc.2011.01.042 PMC3648850

[15] Traggiai E, Chicha L, Mazzucchelli L, Bronz L, Piffaretti JC, et al (2004) Development of a human adaptive immune system in cord blood cell-transplanted mice. Science 304: 104–107. 15064419 10.1126/science.1093933

[16] Isaacson JH, Cattanach BM (1962) Mouse News Letter. 27: 31.

[17] Ito M, Hiramatsu H, Kobayashi K, Suzue K, Kawahata M, et al (2002) NOD/SCID/gamma(c)(null) mouse: an excellent recipient mouse model for engraftment of human cells. Blood 100: 3175–3182. 12384415 10.1182/blood-2001-12-0207

[18] Oshika Y, Nakamura M, Abe Y, Fukuchi Y, Yoshimura M, et al (1998) Growth stimulation of non-small cell lung cancer xenografts by granulocyte-macrophage colony-stimulating factor (GM-CSF). Eur J Cancer 34: 1958–1961. 10023322 10.1016/s0959-8049(98)00236-6

[19] Moro M, Bertolini G, Tortoreto M, Pastorino U, Sozzi G, et al (2012) Patient-derived xenografts of non small cell lung cancer: resurgence of an old model for investigation of modern concepts of tailored therapy and cancer stem cells. J Biomed Biotechnol 2012: 568567. 22547927 10.1155/2012/568567 PMC3324927

[20] Hoffman R (1999) Orthotopic metastatic mouse models for anticancer drug discovery and evaluation: a bridge to the clinic. Invest New Drugs 17: 343–359. 10759402 10.1023/a:1006326203858

[21] Yang M, Baranov E, Jiang P, Sun FX, Li XM, et al (2000) Whole-body optical imaging of green fluorescent protein-expressing tumors and metastases. Proc Natl Acad Sci U S A 97: 1206–1211. 10655509 10.1073/pnas.97.3.1206 PMC15570

[22] Bouvet M, Yang M, Nardin S, Wang X, Jiang P, et al (2000) Chronologically-specific metastatic targeting of human pancreatic tumors in orthotopic models. Clin Exp Metastasis 18: 213–218. 11315094 10.1023/a:1006767405609

[23] Luo S, Zhang E, Su Y, Cheng T, Shi C (2011) A review of NIR dyes in cancer targeting and imaging. Biomaterials 32: 7127–7138. 21724249 10.1016/j.biomaterials.2011.06.024

[24] Zhang X, Bloch S, Akers W, Achilefu S (2012) Near-infrared molecular probes for in vivo imaging. Curr Protoc Cytom Chapter 12: Unit12 27. 10.1002/0471142956.cy1227s60 PMC3334312 22470154

[25] Shu X, Royant A, Lin MZ, Aguilera TA, Lev-Ram V, et al (2009) Mammalian expression of infrared fluorescent proteins engineered from a bacterial phytochrome. Science 324: 804–807. 19423828 10.1126/science.1168683 PMC2763207

[26] Filonov GS, Piatkevich KD, Ting LM, Zhang J, Kim K, et al (2011) Bright and stable near-infrared fluorescent protein for in vivo imaging. Nat Biotechnol 29: 757–761. 21765402 10.1038/nbt.1918 PMC3152693

[27] Maeda H, Bharate GY, Daruwalla J (2009) Polymeric drugs for efficient tumor-targeted drug delivery based on EPR-effect. Eur J Pharm Biopharm 71: 409–419. 19070661 10.1016/j.ejpb.2008.11.010

[28] Goldman JP, Blundell MP, Lopes L, Kinnon C, Di Santo JP, et al (1998) Enhanced human cell engraftment in mice deficient in RAG2 and the common cytokine receptor gamma chain. Br J Haematol 103: 335–342. 9827902 10.1046/j.1365-2141.1998.00980.x

[29] Mizukami Y, Kono K, Maruyama T, Watanabe M, Kawaguchi Y, et al (2008) Downregulation of HLA Class I molecules in the tumour is associated with a poor prognosis in patients with oesophageal squamous cell carcinoma. Br J Cancer 99: 1462–1467. 18841157 10.1038/sj.bjc.6604715 PMC2579690

[30] Vora AR, Rodgers S, Parker AJ, Start R, Rees RC, et al (1997) An immunohistochemical study of altered immunomodulatory molecule expression in head and neck squamous cell carcinoma. Br J Cancer 76: 836–844. 9328140 10.1038/bjc.1997.472 PMC2228257

